# Perceived consequences of extreme risk protection orders: A qualitative study of respondents from California

**DOI:** 10.1371/journal.pone.0361337

**Published:** 2026-10-26

**Authors:** Veronica A. Pear, Christopher E. Knoepke, Lisa B. Geller, Amy Barnhorst, Ari Davis, Rose Kim, Garen J. Wintemute

**Affiliations:** 1 Centers for Violence Prevention, Department of Emergency Medicine, University of California Davis School of Medicine, Sacramento, California, United States of America; 2 Firearm Injury Prevention Initiative, University of Colorado, Aurora, Colorado, United States of America; 3 Center for Gun Violence Solutions, Johns Hopkins University, Baltimore, Maryland, United States of America; University of New South Wales, AUSTRALIA

## Abstract

**Background:**

Extreme risk protection orders (ERPOs) temporarily prohibit the purchase and possession of firearms by people at high risk of harming themselves or others. This study drew on the experiences of ERPO respondents to learn about the perceived short- and long-term consequences of the law.

**Methods:**

For this qualitative descriptive study, we conducted 24 semi-structured interviews between March and November 2024 with respondents to ERPOs issued in California between 2016 and 2020. We purposively sampled 250 respondents for whom we had previously abstracted ERPO court case data to ensure adequate representation across domains of interest (e.g., demographics, location) and invited respondents to participate in the study by mail. All interviews were recorded, double coded, and thematically analyzed.

**Results:**

Our diverse participants described heterogeneous consequences across 4 domains: violence risk, legal/economic outcomes, opinions/attitudes, and social/emotional wellbeing. Two participants believed their ERPOs prevented violence and 1 was unsure; the rest believed that they would not have committed violence irrespective of the ERPO. Some perceived the ERPO to have had negligible effects on their lives, while others described both positive consequences, including self-reflection and behavior change, and negative consequences, including employment or housing difficulties, stigmatization, damaged relationships, loss of trust in legal institutions, and perceived exacerbation of violence risk. Participants reported that they were not connected to mental health care or other supportive services through the ERPO process.

**Conclusions:**

Participants perceived ERPOs to have varied and mixed consequences. These accounts identify outcomes that warrant consideration in ERPO research, policy, and implementation. Future research should assess the prevalence and distribution of these consequences and evaluate whether policy refinements can reduce adverse experiences without compromising the public health benefits of these laws.

## Introduction

In recent years, firearm violence (homicide and suicide) resulted in over 125 deaths per day on average in the US [1] and many more nonfatal injuries. Some of these deaths and injuries are preceded by warning signs that provide an opportunity for intervention, including explicit or implicit threats, paranoia, cognitive decline, and dangerous behavior with firearms. In 22 states, Washington D.C., and the U.S. Virgin Islands, individuals exhibiting these behaviors can be subject to an extreme risk protection order (ERPO), which is a civil order temporarily preventing the purchase and possession of firearms among people judged to be at risk of harming themselves or others.

Details regarding who can petition, order duration, standards of evidence, and more vary between states [2]. In California, law enforcement, immediate family members, roommates, certain individuals at work and school, dating partners, and people who share a child in common can petition a judge for an ERPO, called a gun violence restraining order. Temporary and emergency orders last for 21 days and are followed by a hearing at which the respondent (the subject of the order) can appear. As of September 2020, final orders issued at the hearing can last for up to 5 years; prior to this, they lasted for up to 1 year.

ERPOs are intended to prevent firearm violence by intervening on respondents’ access to lethal means (i.e., firearms). Most suicides (55%) and homicides (79%) involve firearms [1]. In addition, firearms have the highest case fatality rate of any method of suicide or homicide [3,4]. Studies have shown that means restrictions for suicide prevention often do not result in methods substitution [5]. Therefore, removing firearms from high-risk situations should prevent deadly violence.

Indeed, research has found ERPOs to be associated with reductions in suicide at the population level [6,7] and among respondents [8,9]. Associations with interpersonal violence perpetration are more mixed [6,10], but findings from Florida are promising [11].

Nevertheless, this important research has, to date, been limited to violence-related or procedural outcomes conceived of by a researcher and measured with administrative data. This raises the possibility that meaningful consequences of the law have not yet been described, either because researchers have not thought to examine them or because they are not readily measurable with available data. The aim of this study was to explore the heretofore unknown consequences—both positive and negative—of ERPOs by drawing on the experience of those most affected by these laws—ERPO respondents—to learn about the first-hand perceived short- and long-term consequences of the law.

## Materials and methods

This qualitative descriptive study drew on semi-structured interview data from 24 former respondents to ERPOs issued in California. This project was approved by the Institutional Review Boards (IRBs) at the University of California, Davis and Johns Hopkins University.

### Study sample

We used previously abstracted ERPO court case files for orders issued in California, 1/1/2016–6/30/2020, to identify former respondents and their addresses. We took a purposive sample of 250 respondents with the aim of having diversity among participants with respect to race and ethnicity, gender, age at first ERPO (adults and minors), threat type (mass shooting vs other), petitioner type, and location. No ERPO records are automatically sealed in California, including those for minors. We created 5 sets of 50 respondents, with each set having similar distributions of 1) the previously described selection variables, 2) the type of threat precipitating the ERPO (self-harm, interpersonal harm, or both), and 3) final orders. No individuals under the age of 18 when the sample was drawn were invited to participate.

Because the ERPOs were several years old, we first abstracted respondents’ mailing address and identifiers from the court case files and then obtained current mailing addresses from LexisNexis. Recruitment was done sequentially in batches of 50, allowing us to maintain a flexible strategy. We invited respondents to participate via mailed letters containing a $2 bill. Initial letters invited the respondents to participate in a 45-minute semi-structured interview to discuss their experience with and perceptions of ERPOs. Reminders were sent 1, 3, and 6 weeks following the initial invitation. If we did not hear from the respondent after 8 weeks, no further contact was sought. Participation was incentivized with a $100 Visa gift card, which was mailed upon completion of the interview.

Participants could choose to do a recorded Zoom interview or telephone interview with experienced masters- and doctoral-level interviewers with extensive backgrounds in firearm injury prevention (CEK, AB, LBG). Interviewers had no relationship with the participants prior to study commencement. As approved by the IRB, participants provided verbal informed consent prior to beginning the interview, which was documented and witnessed by the interviewer. Interviews were conducted between 3/13/2024 and 11/15/2024.

### Interview protocol

We began by asking participants about their understanding of the ERPO law and its purpose. After this, we asked for details of their ERPO case, such as their relationship (if any) with the petitioner, the general circumstances in their lives when the ERPO was filed (including firearm access), and the precipitating event leading to the order. We then asked participants about their ERPO service experience and the consequences they perceived the ERPO to have had on their lives. We also asked about the ERPO’s perceived appropriateness in their case and whether it prevented violence. Finally, we encouraged the respondents to reflect on their ERPO experience and to offer thoughts about how the law or its implementation could be improved. The full interview protocol is included in the S1 Text. Here, we focus on answers related to perceived consequences of the order, including but not limited to violence prevention.

Interview recordings were professionally transcribed by Datagain and uploaded into Dedoose to facilitate team-based coding.

### Analytic approach

Our codebook was built using a mixed inductive-deductive approach. We first created an *a priori* codebook capturing concepts relevant to the project’s main aims and then refined the codebook after open coding a sample of transcripts as a group. The final codebook was then applied to all transcripts. The study team (VAP, LBG, AD, RK) double coded all transcripts and resolved discrepancies through discussion at standing team meetings. To further enhance trustworthiness, interviewers prepared analytic memos following each interview, which were subsequently discussed by the research team. We conducted a thematic analysis of the coded interview data to describe the respondents’ experiences and identify patterns, similarities, and differences across respondents. Exemplars of each theme were captured directly from the transcripts.

## Results

### Participants

We conducted interviews with 24 former ERPO respondents (13.9% of the 173 former respondents for whom invitations were not returned as undeliverable). Participant characteristics are displayed in Table 1. Sixteen participants (67%) were men, 7 (29%) were women, and 1 (4%) was a trans woman. A plurality of participants were white (42%), followed by Black (29%) and Hispanic (17%). When the ERPO was first issued, participants had a mean age of 36 years (SD: 13 years) and 4 (17%) were minors. Seven of California’s 10 Census Regions were represented, with most participants having orders issued in San Diego-Imperial (29%), the San Francisco Bay Area (21%), or Orange County (17%).

**Table 1:** Participant characteristics.

	Participants (N=24) N (%)
Gender	
Women	7 (29%)
Men	16 (67%)
Trans Women	1 (4%)
Race and Ethnicity	
Asian	1 (4%)
Black	7 (29%)
Hispanic	4 (17%)
Native American	1 (4%)
White	10 (42%)
Unknown	1 (4%)
Age at ERPO, Mean (SD) Years	36 (13)
Minors	4 (17%)
Residential Region at ERPO Issuance^a^	
Superior California	2 (8%)
San Francisco Bay Area	5 (21%)
Central Coast	3 (13%)
Southern San Joaquin Valley	2 (8%)
Los Angeles County	1 (4%)
Orange County	4 (17%)
San Diego-Imperial	7 (29%)

^a^Based on 2020 Census Regions (https://census.ca.gov/regions/)

Basic details about the participants’ ERPOs from the court case files are displayed in **Table 2**. A majority (63%) of participants were first issued an ERPO in 2019. Participants’ ERPOs were issued in response to other-directed threats alone (63%), other-directed threats in conjunction with self-directed threats (25%), and, more rarely, self-directed threats alone (13%). The great majority (92%) of ERPOs were petitioned for by law enforcement. A little under half (46%) of final orders were granted, one-third (33%) were not granted, and the outcome was unknown in the remaining cases.

**Table 2:** ERPO details from court records.

	Participants (N = 24) N (%)
Year First ERPO Issued	
2016	1 (4%)
2017	1 (4%)
2018	3 (13%)
2019	15 (63%)
2020	4 (17%)
Directionality of Threat	
Others Alone	15 (63%)
Self Alone	3 (13%)
Both Self and Others	6 (25%)
Petitioner Type	
Law Enforcement	22 (92%)
Family	2 (8%)
Final Order	
Granted^a^	11 (46%)
Not Granted	8 (33%)
Outcome Unknown^b^	5 (21%)

^a^One participant’s final order was also renewed.

^b^The outcome of the final order hearing was not in the court case file. There was no record of a final order in the California Restraining and Protective Order System, so these were likely not granted.

### Consequences

Some participants perceived the ERPO to have a negligible impact on their lives, stating, “I don’t think the [ERPO] had an impact on my life, to be honest with you” (P4) and the ERPO “doesn’t really affect me in my daily life at all” (P17). Others, however, described a range of positive and negative consequences stemming from the ERPO. These are summarized in Table 3. Consequences fell into 4 broad categories: violence risk, legal/economic outcomes, opinions/attitudes, and social/emotional wellbeing.

**Table 3:** Perceived consequences and associated questions for research and policy.

Category	Consequence	Questions
Violence Risk	• Violence prevention • Exacerbated risk of violence • No connection to services	• Does using ERPOs to connect respondents to needed mental health, substance use, and social services affect respondent experiences or subsequent risk of violence?
Legal/Economic Outcomes	• ERPO on background checks for employment/housing • ERPO used as leverage in other legal proceedings	• Do ERPO expungement processes for former respondents judged to be no longer dangerous affect employment and housing outcomes?
Opinions/Attitudes	• Lack of trust in legal system, law enforcement • Firearm ownership not worth the risk	• Do procedural justice practices influence respondents’ perceptions of legitimacy and trust?
Social/Emotional Wellbeing	• Vulnerability • Social/cultural losses related to losing access to firearms • Stigma • Relationships fractured	• Does the terminology used for ERPO laws influence respondents’ experiences of stigma or public perceptions of respondents?
Other	• No perceived consequences	• N/A

#### Violence risk.

Most (n = 14) participants did not believe the ERPO prevented violence in their lives, not because they went on to commit violence, but because, in their view, the threat of violence was not serious. As one former respondent who was having paranoid delusions when the order was issued stated:

There wasn’t going to be any violence unless someone attacked me and that’s kind of a non-reality. […] The family members that I was afraid of loved me and don’t want to kill me. And so it was all in my head. And so I think that what would happen was a gun would’ve just sat there and nothing would’ve happened. - P19

However, a small number (n = 2) of participants believed the order “definitely” prevented violence (P1) or prevented violence “to a certain extent” (P10) and 1 was unsure. These 3 participants who perceived violence to be at least potentially prevented were issued ERPOs for a range of reasons (including both self- and other-directed threats), and 2 of the 3 were placed on an involuntary psychiatric hold concurrently with the order.

Concerningly, some participants suggested that the ERPO itself precipitated new violence risks. One former respondent who had been issued a final ERPO for threatening his wife and child felt strongly that the order was unjustified and that he was failed by the justice system, which he felt made him more hopeless; he stated:

Honestly, I did think about committing suicide myself because it didn’t make sense, it absolutely did not make sense to me why this would happen in this manner. […] So, it was a very depressing, very difficult, very troubling time for me, and I’m glad, obviously, I didn’t commit suicide. I’m glad I didn’t. But I’d be lying to you today if I said I did not think about committing suicide as a result of that experience. - P12

Another participant, who was issued a final order for making threats to kill his ex-wife’s boyfriend and/or the boyfriend’s children, said the ERPO initially engendered feelings of rejection and apathy that amplified his violent ideation:

I felt like, okay. Everyone’s against me. You know? […] I was like, fuck it. And a little bit like I was kinda going down the dark hole. Like, okay. I don’t give a fuck. They could take my weapons away. I could still... this is America. I can get a gun on... there’s a gun on every block in the fucking America. I could get a gun if I really wanted to get a gun. […] But it was leading me down, you know, pulling me down the slope even more. I don’t give a fuck. You know? I don’t care what happens. - P20

However, participants also described the ERPO as prompting self-reflection and behavior change that reduced their risk of violence. The respondent quoted just above who said the ERPO made him dangerously apathetic went on to say the order ultimately played a positive role in his life: “Yeah, I guess it was a good thing. I don’t wanna say it was, but it helped. But that’s only because the way I took it. You know? Other people could take it and just go down the slope still. I realized I need to turn it around.” Similarly, another participant said the ERPO “was a good thing because I never would have questioned my choices and how I acted if it weren’t for having to face this” (P1).

Though not required, ERPOs could provide an opportunity to connect respondents in crisis to needed services that could help address the underlying issues putting them at risk of violence. The former respondents we spoke to uniformly denied being offered any such services as a result of the ERPO. A majority of participants had co-occurring interventions at the time of the ERPO, mostly arrest and involuntary psychiatric holds. Some of these co-occurring interventions facilitated access to services. For example, respondents described receiving mental health evaluations via psychiatric holds and accessing mental health care and substance use treatment via criminal courts. One participant who was arrested at the time of the ERPO said,

That [being arrested] was probably the best moment of my life. No joke. […] Yeah, best moment of my life, my whole life at that point. I went to jail. […] And then I went to like a local homeless shelter in [city redacted] and then I went to [a rehab facility] in [city redacted] for about almost nine months. And then I went to a safe house for a year. - P14

#### Legal/economic outcomes.

Participants described a range of legal and/or economic consequences of having an ERPO. Several noted that the ERPO appeared on background checks for employment or housing. While some said that this did not impact their career and “didn’t affect [them] getting hired” (P4), others described losing or not attaining employment due to the order. One participant who was having delusions relating to gang stalking at the time of the ERPO lamented, “I had a job with the federal government, because of that restraining order I wasn’t able to go back to. And just a couple of weeks ago, I applied for a position as a substitute teacher. Because of that restraining order, I wasn’t able to obtain that job” (P18). Another participant, who was issued a temporary order for threats of self-harm, felt the order negatively impacted her employment and housing due to its appearance on a background check:

When I’ve applied for rentals, it’s come up when people have done backgrounds. And I’ve had people refuse to rent to me because of it. […] I applied for a victim advocate job. It was for children because I thought okay, I can make a difference for kids. […] I thought that I could make a difference and then once [the ERPO] comes up they’re like, no, you can’t be around kids. And it’s like, no, but it didn’t even go through. The judge threw it out. So […] I can’t even do what I care about because I’m looked at as this bad person or scary person or unsafe person.” - P8

A few participants carried firearms for work when the order was issued, with varying consequences on their employment. An armored truck driver was moved to a different task not requiring a firearm for the duration of the order. Others lost employment and careers: one participant issued an ERPO for suicide threats was in law enforcement when the order was issued and claimed that the ERPO resulted in her losing her job and “made it so [she] can’t work in the law enforcement field anymore” (P8). Another was working as a security guard and “couldn’t get a job anywhere” (P16) after the ERPO was issued (although he was also charged with felony criminal threats, which likely played a role).

The other legal/economic consequence that participants described was the perception that ERPOs were being used as leverage in other legal proceedings. One former respondent described this positively, as his ERPO was part of a plea agreement that resulted in his felony charges being reduced to misdemeanors: “After I plead guilty, then it was explained to me that since I plead guilty, these orders are going to be put in place, which happened to be the [ERPO] taking away my rights to own a firearm” (P3).

Three participants, all men issued orders in response to threats of violence against family or current or former intimate partners, believed the orders were used as leverage against them in other court cases, usually a divorce. Two of these respondents felt that the ERPO biased judges against them: “Even though I was found not guilty [for the final order], they can still tell the judge that that [ERPO] happened, and then there’s still that bias, right?” (P4). Similarly, another participant noted “Every single time something came up, the response was, oh, well, don’t forget he was already found guilty of the [ERPO]. So, the judge is going to know that and know that he’s a threat and all this other stuff” (P12). Another participant believed that his ex used the ERPO “to get custody of [his] daughter” (P9). ERPOs were also perceived to have harmed participants in subsequent criminal trials: “And now it’s also affecting me because of this new case that I have [involving firearm charges]. Since I had priors with it, they’re like, oh, these are his guns. The previous charges, and now he has to take these charges” (P15).

#### Opinions/attitudes.

Participants described the ways in which their ERPO experiences shaped or changed their thoughts, beliefs, opinions, and attitudes about the legal system, law enforcement, and firearm ownership. One participant described how the ERPO resulted in his loss of faith in the legal system:

I felt like the justice system failed me. […] As a result of that experience, I don’t have any trust for our justice system, I don’t have any trust for judges. I don’t trust. […] I question law enforcement’s motive. I question the judge. I question the court system, like all of that, I feel like, has been compromised for me unfortunately. […] My trust of the whole system has been compromised, unfortunately. - P12

Another described feeling like the system was rigged against him, as a former felon facing a law enforcement petitioner: “The judge will [say], ‘I don’t care what you’re saying because you’re a felon. He’s a law enforcement officer. I’m going to believe the law enforcement officer over a felon any day’” (P23).

While some participants described law enforcement officers as “good people” (P20) who were “just there to do their job” (P4), others described being scared and mistrustful of law enforcement as a result of the ERPO experience. This latter group comprised individuals belonging to socially vulnerable populations, including women, minors (at the time of the order), and Black and Brown individuals. One participant, a white woman, said, “after I was served, I became very scared of police officers, and that continues” (P1). A Hispanic man expressed losing trust in law enforcement following the ERPO: “Now I don’t trust them at all” (P15). This sentiment was shared by a Black woman who said she had “no respect for law enforcement” because of “what they said, what they did, how they did it and what they wrote on the [ERPO] form. No respect. I don’t even trust them” (P18). A former minor respondent said his “whole perspective of law enforcement kind of flipped” because he perceived the officers in his case to be “pushing paper” rather than seeking the truth of “who’s the bad guy and who’s the good guy” (P6).

Participants’ experiences with ERPOs also influenced their thoughts about firearm ownership. One participant who was a minor when the ERPO was issued noted that he learned he “probably shouldn’t own a gun” (P24). He went on to explain, “The statistics of firearms where it’s like, you’re most likely to die by your own firearm than anybody else’s if you own one. That’s the most likely scenario.” Other participants similarly expressed the idea that firearm ownership was not worth the risk, but for different reasons; these individuals were dissuaded by the presence of firearm laws: “So I feel like me being in California with these kinds of laws, I don’t even want to have my Second Amendment. Because I don’t want to be arrested” (P21). Similarly, a former respondent who immigrated to America noted: “My wife was upset and told me that she didn’t want me to have guns again, that by having guns I had gotten into trouble […]. Now that I see it, I would never buy a firearm again” (P11). This influence also extended to family and friends who were privy to the participants’ ERPO experiences: “No one in my family wanted to get [a firearm], especially after this whole incident. They were especially, like, doubly not going to get one. No one around me that was still friends with me at the time wanted to get one either” (P2). It is worth noting that while several participants expressed not wanting to own firearms anymore, others noted that they successfully obtained their legally removed firearms or purchased new firearms after the ERPO expired, with one “mark[ing] the date on [his] calendar” to ensure his firearms would be returned as quickly as possible (P12).

#### Social/emotional wellbeing.

In addition to changes in beliefs and attitudes, participants also described emotional and interpersonal consequences of the ERPO, including vulnerability, stigma, and loss of relationships. Some participants described losing access to firearms as having negative social and emotional consequences. Several felt that their “sense of safety” (P3) was compromised without firearms, which were described as “my source of protection” (P19) and “the only thing I had to feel like I had some security” (P8). As one veteran explained: “I feel more secure with the weapon, with the AR, because that’s what I was trained on when I was in the Army, you know. That’s what was a part of me for years. So I feel like a little, like, vulnerable on the streets when I go out on the streets. […] I feel safer with the weapon on me” (P20). This participant went on to clarify the limited impact of not having access to a firearm: “At the same time, I feel pretty good. […] I would feel better with the weapon on me, and I feel okay without having it on me.”

Participants also described the social and cultural impact of losing their firearms. For example, one participant missed out on his family’s annual hunting trip while he was prohibited. Another, who had several diagnosed mental health disorders and was issued an ERPO for threats of self-harm, described the socioemotional impact of no longer going to the shooting range:

I just had fun going to the range and then being able to be part of that community where we would get together. […] So that was a way for me to connect with other people, which I wasn’t really doing on a daily [basis] because I was always at work doing something at work or going to school. So that was like my go-to coping skill. So when that got taken away, I had to learn some new ones. - P22

Other social and emotional consequences of the ERPO included stigma from being a respondent and damage to relationships as a result of the precipitating event and ERPO process. A respondent who was issued an order for threats of self-harm alone described how people “suddenly act afraid of me because they’ve seen it” on a background check (P8). She went on to say:

They see the [county] versus [name] gun violence restraining order and they don’t know the details. So they just think that title makes it sound like you’re gun violent. […] I’ve had a lot of people look at me like I’m a monster because of it. […] But it’s hard that I’m put in a situation where I have to defend myself, and where people just automatically think awful things when they don’t know the situation. - P8

This sentiment was echoed by another respondent, who noted that you look “like a psychopath when you have [an ERPO]” (P16).

Other respondents described the ERPO as fracturing their relationships. One former respondent stated the ERPO played a role in her divorce, as she blames her husband for working with law enforcement to obtain the order. She explained: “When I saw the video of the police cam of him saying, ‘Yeah, just make sure you take her out the house. Take the gun. Don’t let the gun be in the house. She’s crazy. She’s hallucinating.’ I mean, how can you actually say you love me and you do this to me?” (P18). Another participant who was issued an ERPO as a minor after making a school shooting threat said he “had no one else” after his friends “all left” him after “they heard the whole situation” (P6).

## Discussion

Former ERPO respondents perceived a wide range of short- and long-term consequences to stem from the order, both positive and negative, reflecting their nuanced and varied experiences. Broadly, consequences were related to violence risk, legal/economic outcomes, opinions/attitudes, and social/emotional wellbeing. Some reported benefits of being a respondent to an ERPO, such as perceived violence prevention, self-reflection and behavior change, and being able to leverage the ERPO for a reduced sentence in related criminal cases. Others described perceived harms, including new violence risks, employment or housing trouble as a result of the order appearing on a background check, mistrust in the legal system, and stigmatization. These findings highlight the heterogeneity of respondents’ experiences and suggest that ERPOs may have consequences extending beyond firearm access and violence outcomes.

Two of our 24 participants believed the ERPO prevented them from harming themselves or others, and another thought that it might have prevented violence but was not sure. Most did not believe that violence had been prevented, generally because they did not view themselves as likely to have acted on the threats precipitating the order. These retrospective assessments cannot establish what would have occurred in the absence of the order. Participants who perceived that violence was prevented may have attributed subsequent nonviolence to the ERPO even if violence would not have occurred otherwise, while those who perceived no benefit may have underestimated the seriousness of the preceding risk. Social desirability, recall, and self-evaluation bias may have shaped both types of accounts. Accordingly, these data describe respondents’ perceptions of violence prevention but cannot establish whether or how often the orders prevented self-directed or interpersonal violence.

Concerningly, a few respondents mentioned that the ERPO itself exacerbated their risk of violence. One described new suicidal ideation, while another described intensified interpersonal violence ideation driven by feelings of apathy and hopelessness. These accounts indicate that an intervention intended to reduce violence risk may, in some circumstances, be experienced as contributing to perceived risk. Further research is needed to identify how common this is, whether these experiences are specific to ERPOs or a reaction to legal system involvement more broadly, and to identify factors that may contribute to or mitigate these experiences.

Participants uniformly reported that the ERPO process itself did not connect them to healthcare or other services, though some accessed behavioral health care through co-occurring interventions like mental health holds or criminal court proceedings. While not required, ERPOs present an opportunity to connect respondents to services and systems that may help the respondent address the underlying causes of the crises leading to the orders. Some states, like Washington and Colorado, permit judges to order mental health evaluations as part of the ERPO; however, these evaluations are distinct from ongoing, voluntary care, and less than half of Washington’s ERPOs include court-ordered mental health evaluations [12]. A study of ERPO court case files in California during the law’s first three years of implementation found that ERPO respondents were reported to have mental illness, substance use, employment problems, and housing instability [13]. This population could potentially benefit from receiving a range of healthcare and social services—in conjunction with the firearm prohibition—aimed at addressing these underlying contributors to risk. Whether and under what conditions offering behavioral health or social services through the ERPO process improves respondents’ experiences or reduces subsequent self-directed or interpersonal violence remains unknown and should be evaluated empirically.

Another concerning consequence of ERPOs described by several participants was employment or housing difficulty when the ERPO appeared on a background check. The impact of ERPOs on employment likely depends on many factors, including whether firearm carriage is an occupational requirement, characteristics of the job, and the degree of discretion exercised by the hiring manager. Although ERPOs are civil rather than criminal orders, research demonstrates that the presence of a criminal record on background checks is associated with worse employment outcomes for Black compared with white job seekers [14], highlighting how discrimination and biases compound for people with multiple marginalized identities. Our study was not designed to rigorously test for differences in perceived consequences by race, but this is an important direction for future work. Additionally, given that economic stress is a risk factor for violence perpetration [15,16], future research should examine the frequency, duration, and distribution of these consequences and assess whether targeted employment or housing assistance can mitigate them.

Participants’ accounts also raised questions about how long a temporary civil order should remain visible on public-facing records after the order expires. In 2025, Indiana amended its ERPO statute to allow former respondents to request to have the order expunged from their record, provided that the court finds the respondent to be not dangerous [17]. This approach provides one model for balancing respondents’ concerns about continuing consequences with public safety considerations. Research is needed to assess how such provisions operate in practice, including their accessibility, use, and effects on employment, housing, and safety.

Respondents also felt stigmatized by the ERPO appearing on background checks or when others inferred from the order’s name that they posed a risk of interpersonal violence. Stigma has been linked to anxiety, depression, and social withdrawal in criminal offenders [18,19]. Participants’ accounts suggest two policy features that warrant further examination: the duration and public visibility of ERPO records and the terminology used to name the orders. As described above, policies allowing eligible former respondents to have the ERPO expunged could potentially provide the respondents with a path to limit the duration for which the order would appear on background checks. In addition, names that do not imply that respondents have threatened interpersonal firearm violence would more accurately reflect the range of cases for which orders are used and have the potential to reduce inaccurate and stigmatizing inferences about the nature of the crisis leading to the order. “Risk protection orders” (Florida and Connecticut) and “firearms restraining orders” (Illinois) are examples of alternative terminology in current use.

Finally, several former respondents expressed losing trust in the legal system or law enforcement as a result of their ERPO experience. Such legal cynicism may result in individuals being less likely to call the police in an emergency or to cooperate with police in an investigation [20,21]. However, research in other contexts suggests that procedural justice may strengthen perceptions of legitimacy and trust in the legal system and law enforcement [21]. In the context of ERPO laws, potentially relevant practices include prompt notification of the final order hearing and clear information about firearm storage and return. Colorado’s and Maine’s policy of providing ERPO respondents with an attorney [22,23] may also improve procedural justice, particularly when the petitioner is a law enforcement agency that may be represented by a city or district attorney. Whether such approaches would address the concerns raised by ERPO respondents remains an open question.

Understanding the lived experience of ERPO respondents can help researchers identify the range of outcomes that might stem from this policy, including those that are unanticipated or unintended, and can help inform policy refinements to maximize the benefits of ERPO laws while minimizing their negative consequences. Prior studies have found ERPO laws to be associated with reductions in suicide among respondents and at the population level [6,7,9], with some evidence of reductions in population-level firearm homicide or assault [11,24]. These public health consequences should be considered alongside the individual consequences described by respondents here. This study demonstrates that ERPO respondents have heterogeneous experiences, and that these experiences constitute an additional and meaningful dimension of ERPO evaluation. We note that findings reflected in our data represent only consequences experienced by respondents and are necessarily a partial reflection of the full range of ERPO consequences. Additional research incorporating the experiences of petitioners, family members, those threatened by the respondents, and those serving the orders is needed to develop a fuller understanding of the benefits, harms, and tradeoffs associated with these laws.

### Limitations

This study is the first to explore the first-hand perceived consequences of being a respondent to an ERPO. It benefits from a diverse sample of former respondents, including a disproportionately large number of women and people of color compared to the overall demographics of ERPO respondents in California [25]. Nevertheless, it is subject to limitations. Results are based on interviews with only 24 former respondents, and participants are unlikely to be representative of respondents in California or elsewhere. Participants may be more likely to feel strongly—whether positively or negatively—about the ERPO law and their experience with it than those who chose not to participate. Respondents were also reflecting on events that occurred several years ago; this allowed for longer-term consequences to develop and for the intense emotions surrounding the order to subside but may also have contributed to recall bias. Another limitation is that it was often not possible for the respondents to disentangle consequences stemming from the ERPO from those stemming from other co-occurring interventions (e.g., arrest or involuntary psychiatric holds), which were common. Participants were often unclear about the complex causal chains of events that branched from an intense and chaotic time in their lives. We did our best to present the consequences that participants linked to the ERPO while omitting those that were more likely to stem from other related interventions (e.g., criminal convictions).

## Conclusions

This qualitative descriptive study drew on semi-structured interview data from 24 former ERPO respondents in California to characterize the perceived short- and long-term consequences of the law on those it directly affects. Participants described both positive and negative consequences stemming from the ERPO. Some believed that the orders prevented violence and served as an impetus for self-reflection and behavior changes, while others described new violence risks, employment problems, and stigmatization. These findings can inform refinement of ERPO policies and their implementation, though whether such policy changes effectively prevent or reduce the potential adverse consequences described here remains unknown. They also highlight the complexity of respondents’ experiences and suggest that evaluations of ERPOs should consider a broader range of outcomes than violence prevention alone.

## Supporting information

S1. Text ERPO Respondent Interview Protocol. (PDF)
