# Supplementary material for: Perceived consequences of extreme risk protection orders: A qualitative study of respondents from California
Source: PLoS One. 2026 Oct 26;21(10):e0361337. doi: 10.1371/journal.pone.0361337 (PMC13704857; doi:10.1371/journal.pone.0361337)
Supplement: Supplementary file 1 [file pone.0361337.s001.pdf]

S1 Text: ERPO Respondent Interview Protocol

Perceived Consequences of Extreme Risk Protection Orders: A Qualitative Study of Respondents from California

Pear VA, Knoepke CE, Geller LB, Barnhorst A, Davis A, Kim R, Wintemute GJ

Just to confirm, I understand that you were involved with a gun violence restraining order in [month and year]. Is that correct?

If yes, obtain informed consent and start recording.

Before we get started, I first want to ask about where you are calling from. Are you in a safe place right now? What city are you in? *If it's not obvious:* And what county is that in?

We're so thankful that you agreed to talk with us today. We understand that this may have been a stressful time in your life and that it may be hard for you to talk about. Can you tell me why you want to participate in the study?

We're going to start with some general questions about GVROs.

1) General GVRO questions

a) What is a GVRO, as you understand it?

i) What is the purpose of a GVRO?

ii) Under what circumstances should they be used?

  b) Had you ever heard of a GVRO before you were the subject of one?
    i) From where? (Advertisement? Word-of-mouth? From a law enforcement/mental health/other professional? Other?)

Now I'd like to ask about the GVRO case that you were involved in.

2) Petitioner information

  a) Do you know who the petitioner was?
    i) *If YES, and the petitioner was not a law enforcement officer:*
      (1) **What was your relationship with [the petitioner]?**

        (2) Did you live together at the time of the GVRO petition? Do you live together currently?
        (3) *If not an immediate family member:* How long have you and [the petitioner] known each other?
    ii) *If NO: Do NOT reveal the petitioners name to the respondent.*
  b) *If petitioner is known to respondent:* How would you like me to refer to this person during our conversation? Would you like me to use his/her first name, refer to him/her as your [*insert relationship from above*], or in some other way?

Let's talk, in general, about the days just before the GVRO was filed.

3) <u>General circumstances giving rise to the GVRO, including role of firearms</u>
  a) Tell me about yourself in the days just before the GVRO. What was going on in your life?
    i) Describe what a typical day was like for you at that time.
      (1) How did you spend your time?
        (a) Working/going to school/doing something else?
      (2) Describe where you were living. Were you living there alone or with other people?
      (3) *If petitioner is known to respondent and is not a law enforcement officer:* At that time, what was your relationship with [the petitioner] like?
  b) Was there anything you consider out of the ordinary going on with you or in your life at that time?
    i) Were there any major changes in your life that happened around this time?
    ii) Was there anything causing you an unusual amount of stress?
    iii) Thinking back, is there anything that stands out to you about that time in your life?
  c) Describe how you were feeling, physically and emotionally, at that time.
    i) How many hours of sleep would you get in a typical night?
      (1) How was your ability to fall and stay asleep?
      (2) How was your energy when you were awake?
    ii) Describe your appetite and eating habits.
    iii) What about your alcohol or drug use?
    iv) Describe your mood at that time.
      (1) How reactive were you to positive or negative events?
      (2) How was your motivation, interest, or enthusiasm?
      (3) How often did you feel agitated or irritated? Anxious? Angry?
    v) How was your ability to work/study/function?
    vi) Was this unusual, or would you describe your sleep, eating, substance use, and mood during this time as normal for you?
    vii) How was your physical health around the time of the GVRO?
  d) Thinking back to this time, did anyone in your life indicate that they were worried about you?
    i) *If YES:*
      (1) Tell me about that.

(2) Why do you think they were worried?

e) **Has a healthcare or mental health care professional ever told you that you have a mental health or emotional condition?**
   (1) When was this? (Before or after the GVRO?)
   (2) Do you have a family history of mental health or emotional conditions?

f) In the days just before the GVRO was served, did you have access to firearms?
   i) **Did you personally own any firearms?**
      (1) *If YES*:
         (a) How many firearms did you own?
            (i) What types of firearms did you own?
            (ii) Did you acquire any firearms in the 6 months before the GVRO?
               1. *If YES:* How long before the GVRO did you acquire these firearms?
            (iii) For about how long have you owned guns?
               1. What are your reasons for owning guns?
         (b) How did you acquire your guns?
            (i) Did you buy them, inherit them, get them as gifts, or get them another way? What other way?
         (c) How were you storing your firearms?
            (i) Where were you keeping them? (For example, in your house, car, garage, or some other place.)
         (d) Were any of them stored unlocked and/or loaded?
      (2) *If NO:*
         (a) Were you planning to purchase a firearm?
         (b) Were you in the firearm purchase waiting period?
   ii) Did you personally own any ammunition?
      (1) *If YES:*
         (a) How much ammunition did you have?
         (b) Did you acquire ammunition in the 6 months before the GVRO?
            (i) *If YES:* How long before?
         (c) Was there anything out of the ordinary about your ammunition purchases in the 6 months before the GVRO?
   iii) **Did you have access to someone else's firearms?**
      (1) *If YES:*
         (a) Whose firearms did you have access to?
            (i) Did they live with you?
         (b) How many firearms did you have access to and what type were they?
         (c) Were any of them stored unlocked and/or loaded?

Now I'd like to ask you about the specific events that led to the GVRO.

4) Precipitating event
   a) **What's your understanding of why [the petitioner] filed for a GVRO? (What were the reasons?)**

i) *If non-LEO petitioner:* Did [the petitioner] talk to you directly about these things at the time?
ii) Do you think, in general, a GVRO is appropriate in those circumstances?
b) Do you think there were valid reasons for filing the GVRO in your case?
i) Did you harm or threaten to harm yourself or others?
(1) Had you harmed yourself or others in the past?
c) *If LEO petitioner:* Do you know how and why law enforcement got involved?
i) Do you know who contacted law enforcement?

Let's talk about what it was like for you when the GVRO was served.

5) <u>Experience having the GVRO served</u>
a) Describe what happened when you were first served the GVRO.
i) Where were you?
(1) Were you at home? Were you in court? Were you somewhere else?
ii) What were you doing?
iii) Was anyone else with you?
iv) Who served the order to you? Was it a law enforcement officer?
(1) How long did it take for the person to serve the order? How much time elapsed between when the server approached you to when they left?
(2) How many officers were there? Where there other people involved with service that were not officers?
b) Did you find out that you were the subject of a GVRO before being served?
i) How did you find out?
c) How did you respond when you were served the GVRO?
i) Do you remember what you said or did in immediate response to the order?
d) How did you feel when you were served the GVRO?
i) On a scale of 1 to 5, with 1 being not angry at all and 5 being extremely angry, how angry were you when you were served the GVRO?
(1) Why did you feel that way?
(2) Did you yell, throw things, or otherwise express your anger?
ii) On a scale of 1 to 5, with 1 being not surprised at all and 5 being extremely surprised, how surprised were you when you were served the GVRO?
(1) Why did you feel that way?
iii) Did you feel afraid?
(1) Why did you feel that way?
(2) What would have made you feel less scared?
iv) What else were you feeling?
e) What happened when the law enforcement officers who served you the GVRO asked for your guns?
i) How did you feel about being asked to turn over your guns to these officers?
ii) Did you let them have your guns?
(1) Did they get a warrant to search your house for guns?
(2) About how many of your guns were removed as a result of the GVRO?

f) **Do you think you were you treated fairly by [the officers/people/person] who served you the GVRO?**

Let's move on now to what happened in your life after the GVRO was served.

6) Experience after the GVRO was served
   a) What did you do immediately after the person who served you the GVRO left?
   b) **What were the immediate and long-term consequences of the GVRO on your life?**
      i) Did anything about how you were feeling or behaving change in the following days?
      ii) What about now? Do you feel or act differently now versus how you felt at the time the order was served?
   c) Did you receive any health, mental health care, or substance use services as a result of the GVRO?
      i) What kind of services?
      ii) Did you receive these services voluntarily?
   d) Did you see or speak to [*insert name of petitioner, if known to respondent*] after you were served the order?
      i) Has your relationship with [*insert name of petitioner, if known to respondent*] changed as a result of the GVRO?
   e) Do you think the GVRO was overall a good thing or a bad thing?
      i) Were there positive or negative consequences that you didn't expect?
         (1) Are you still feeling these consequences? If not, how long did they last?
         (2) Do you feel like firearm violence was prevented?
      ii) Was it a good thing or a bad thing for those around you?
   f) Was there a court hearing to extend the GVRO beyond 21 days?
      i) Did you attend?
      ii) Were you represented by a lawyer?
   g) Is this GVRO still in effect?
      i) *If YES*: Do you know how long the order will stay in effect?
      ii) *If NO:* When did the GVRO expire?
   h) Did you requested to have the GVRO terminated early?
   i) **Did you request to get your guns back**?
      i) *If YES:* What did you do to request them?
         (1) Did you get them back?
            (a) *If YES:* How long did it take?
               (i) What were the steps in getting them back?
            (b) *If NO:* Why didn't you get them back?
      ii) *If NO:* Do you want to get your guns back?
         (1) *If YES:* Why did you not request to get them back?
         (2) *If NO:* Why not?
            (a) Do you feel safer without them?
   j) Have you purchased any firearms since the GVRO expired?
      i) *If YES:* How many? Why did you purchase them?

Finally, I'm going to ask you to reflect on your experiences and the GVRO law as a whole.

7) Miscellaneous
   a) How much would you agree with the following statement: "Gun violence restraining orders save lives." Would you say you strongly agree, agree, disagree, strongly disagree, or aren't sure if you agree or disagree?
      i) Why?
      ii) Do you think you might feel differently if you had not been the subject of a GVRO?
   b) Based on your experience, do you have any advice for other respondents, petitioners, or those who might serve a GVRO?
      i) How do you think the GVRO law could be improved?
   c) What effect did the GVRO have on your relationship with or opinions about law enforcement?
      i) Do you generally trust law enforcement? Do you think they try to protect you and your community from harm?
      ii) *If served by law enforcement*: What were your feelings towards the officers who served you the order at the time of service? What about now?

8) Interviewee comments
   a) Is there anything I haven't asked about that you'd like to tell me?
   b) Do you have any questions for me?

9) Closing
   a) *Thank the respondent for their participation and time.*
   b) Would you be willing to speak with us again if we have follow-up questions?
      i) ***If yes:*** **What is the best way to contact you in the future?**
         (1) Are you willing to share a friend or relative's phone number? We will only contact them if we can't reach you at the number you provided.
   c) *Give the respondent our contact information: [redacted]*
